# Notes and Notices

**Published:** 1893-05

**Authors:** 


### NOTES AND NOTICES.

LITERARY NOTE.—The value and utility of that unique literary publication, *The Weekly Bulletin of Newspaper and Periodical Literature*, published at 5 Somerset Street, Boston, are to be greatly enhanced by the immediate addition of some important new features. Besides serving as a guide and index to the press of the country by affording a weekly classified and descriptive catalogue of the contents of over twelve hundred different papers and magazines, the *Bulletin* will hereafter supply the growing public demand for a review of the periodical press by devoting several pages every week to comprehensive summaries of the best and most interesting articles appearing in the monthly magazines and the daily and weekly papers.

"CHILDHOOD" is the name of a new monthly magazine, edited by Dr. George William Winterburn. It covers a field not hitherto occupied, being *for* parents and *about* children. It contains thirty-two double column pages, and is sold at the small price of ten cents a number, or $1 a year. It is believed that there are a very large number of parents who desire to give their children the advantage of the best training. *Childhood* will attempt to be the guide of such, and by presenting the subject in all its phases, by means of short, well-written contributions, to supply information which cannot be found elsewhere.

DR. HERBERT BEALS has removed his office to No. 176 Franklin Street, Buffalo, New York.

DR. CLARENCE M. SELFRIDGE has removed his office and residence to 1426½ Geary Street, near Laguna, San Francisco, Cal.

DR. C. EURICH has removed from 124 East Eighty-fifth Street to 209 East Eighty-seventh Street, four doors below Third Avenue, New York City.

GRACE HOSPITAL, DETROIT.—The next regular competitive examination for position of Junior Assistant to the House Surgeon of the Grace Hospital, Detroit, will be held at the Hospital on Saturday, June 24th, at 4.30 P. M. Term, eighteen months. First six months as Junior Assistant; second six months as Senior Assistant and Ambulance Surgeon; third six months, House Surgeon.

Applicants must show evidence of graduation from a recognized Homœopathic college.

All applications must be sent to the President of the Medical Board, The Grace Hospital, Detroit, Mich., not later than June 15th, accompanied by a certificate of good moral character.

CHICAGO HOTEL RATES.—Dr. J. S. Mitchell, Chairman of World's Congress of Homœopathic Physicians and Surgeons, desires us to announce that hotel rates have *not* been raised in Chicago during the Exposition period. Those who do not desire to pay the usual rates of first-class hotels can find very reasonable accommodations by addressing Dr. A. K. Crawford, 70 State Street, Chicago.

THE KLIP.—The advertisement of this invaluable appliance for every physician who wishes to preserve his pamphlets continues in our pages. The klip is highly recommended by the editor of this journal.

